# Resistant and refractory hypertension: two sides of the same disease?

**DOI:** 10.1590/2175-8239-JBN-2018-0108

**Published:** 2018-12-06

**Authors:** Elizabeth Silaid Muxfeldt, Bernardo Chedier, Cibele Isaac Saad Rodrigues

**Affiliations:** 1 Universidade Federal do Rio de Janeiro Faculdade de Medicina Pós-Graduação em Clínica Médica Rio de JaneiroRJ Brasil Universidade Federal do Rio de Janeiro, Faculdade de Medicina, Pós-Graduação em Clínica Médica, Rio de Janeiro, RJ, Brasil.; 2 Universidade Estácio de Sá Curso de Medicina Rio de JaneiroRJ Brasil Universidade Estácio de Sá, Curso de Medicina, Rio de Janeiro, RJ, Brasil.; 3 Pontifícia Universidade Católica de São Paulo Faculdade de Ciências Médicas e da Saúde Departamento de Medicina Rio de JaneiroRJ Brasil Pontifícia Universidade Católica de São Paulo, Faculdade de Ciências Médicas e da Saúde, Departamento de Medicina, Sorocaba, SP, Brasil.

**Keywords:** Resistant Hypertension, Refractory Hypertension, Sympathetic Nervous System, Hyperaldosteronism

## Abstract

Refractory hypertension (RfH) is an extreme phenotype of resistant hypertension (RH), being considered an uncontrolled blood pressure besides the use of 5 or more antihypertensive medications, including a long-acting thiazide diuretic and a mineralocorticoid antagonist. RH is common, with 10-20% of the general hypertensives, and its associated with renin angiotensin aldosterone system hyperactivity and excess fluid retention. RfH comprises 5-8% of the RH and seems to be influenced by increased sympathetic activity. RH patients are older and more obese than general hypertensives. It is strongly associated with diabetes, obstructive sleep apnea, and hyperaldosteronism status. RfH is more frequent in women, younger patients and Afro-americans compared to RFs. Both are associated with increased albuminuria, left ventricular hypertrophy, chronic kidney diseases, stroke, and cardiovascular diseases. The magnitude of the white-coat effect seems to be higher among RH patients. Intensification of diuretic therapy is indicated in RH, while in RfH, therapy failure imposes new treatment alternatives such as the use of sympatholytic therapies. In conclusion, both RH and RfH constitute challenges in clinical practice and should be addressed as distinct clinical entities by trained professionals who are capable to identify comorbidities and provide specific, diversified, and individualized treatment.

## Introduction

Resistant hypertension (RH) has been studied in several different populations since the end of the 20^th^ century. Nevertheless, it was only in 2008 that the American Heart Association published the first guidelines on RH, standardizing its definition and establishing the main risk factors, secondary causes, and the diagnostic and therapeutic approach to these patients^1^. Thenceforth, many studies have demonstrated the high cardiovascular morbidity and mortality and begun to advocate for new therapeutic regimens (i.e. adding definitively spironolactone as the fourth-line drug choice), as well as new interventional therapies seeking for a better blood pressure (BP) control.

In an effort to define a subgroup of high-risk patients who should benefit the most from these new therapies, the refractory hypertension (RfH) definition was established in 2012^2^ for individuals with worst BP control and, possibly, the worst cardiovascular outcomes.

Despite the final common pathway of an increased sympathetic tonus and hydrosaline retention, the current literature suggests the existence of different clinical phenotypes with different prognoses. These phenotypes would range from arterial hypertension that is responsive to initial treatment to RH and, more recently, to RfH.^2^

Although RfH seems to be an extreme phenotype of RH, recent studies have suggested different pathophysiological mechanisms. Whereas an increased sympathetic activity plays a more important role in the former, inappropriate hydrosaline retention due to a renin-angiotensin-aldosterone system (RAAS) hyperactivity is a major factor in RfH.^3^^-^6 Therefore, although hypertension is frequently understood as part of a *continuum*, a better comprehension about the prevalence of RfH in different populations, as well as its clinical and prognostic differences from RH is essential, especially in the post-mineralocorticoid-antagonist-receptor era^7^^-^9.

## Discussion

### Definition

RH is defined as an office BP that remains above the goal despite the use of 3 or more anti-hypertensive agents of different mechanisms of action at optimal doses, preferentially including a diuretic agent. Patients with controlled office BP on 4 or more drugs are also considered RH.^1^^,^^10^

In parallel to this, the definition of RfH has been evolving since 2012,^2^ being currently regarded as the failure of office BP control despite the use of 5 or more anti-hypertensive agents including a long-acting thiazide-like diuretic (ideally chlorthalidone) or a loop diuretics, according to estimated glomerular filtration rate (eGFR), besides a mineralocorticoid receptor antagonist (e.g. spironolactone) as the fourth drug.^7^

### Epidemiology

The prevalence of RH, as estimated by multiple multicenter cohorts, lies between 10-20% of all treated individuals.^11^^-^14 The increased prevalence, despite the improvement of anti-hypertensive regimens in the last 30 years, is explained by the progressive ageing of the population and by the obesity pandemic.^15^ Analyses excluding pseudo-resistant hypertension are needed to estimate the true prevalence of RH.^8^^,^^16^ In Brazil, the Brazilian Longitudinal Study of Adult's Health (ELSA) found an 11% RH prevalence among a cohort of more than 15,000 individuals between 35 and 74 years old.^17^

The prevalence of RfH has been estimated by a limited number of studies. Of particular importance, is the prospective analysis conducted by Dudenbostel,^18^ which reported a 5% prevalence of RfH among the RH referred to a specialized hypertension clinic. Additionally, in the REGARDS study,^19^ similar rates (3.6%) have been described among patients with controlled or uncontrolled RH, highlighting the low prevalence of RfH (0.5%) among the entire population of hypertensive patients. Recently, the analysis of a Spanish ABPM Registry evidenced a prevalence of 8% of RfH among the RH patients (16.9%).^20^

### Mechanisms

RH is mainly attributed to RAAS hyperactivity and consequently to excessive hydrosaline retention, as evidenced by BP reduction with diuretic therapy that is proportional to effective intravascular volume depletion.^21^ This mechanism appears to be multifactorial, being associated with increasing age, obesity, chronic kidney disease (CKD) and diabetes, Afro-American ethnicity, excessive sodium intake, and, remarkably, to the magnitude of the hyperaldosteronism status.^22^

In contrast, RfH would be less volume-dependent, since, by definition, its treatment with the association of diuretic drugs fails to achieve the BP goals.^4^^,^^5^ Thus, refractory hypertensives seem to be under a greater sympathetic influence, having lower levels of plasmatic aldosterone and a reduced 24-hour sodium excretion. Recent studies comparing patients with refractory to resistant hypertension have shown increased markers of sympathetic activity in the former group: higher heart rate, increased 24-hour norepinephrine excretion, and a higher peripheral resistance.^4^^,^^18^^,^^23^

### Clinical characteristics and comorbidities

Resistant hypertensives tend to be older, overweight, or obese. Commonly associated comorbidities include CKD, diabetes, obstructive sleep apnea, left ventricle hypertrophy (LVH), cardiovascular and cerebrovascular diseases and, lastly, hyperaldosteronism status.^1^^,^^10^^,^^11^^,^^24^

Refractory patients, compared to their controlled resistant counterparts, are more likely to be younger, Afro-american, and, predominately, females.^21^ Regarding associated comorbidities, the most common are heart failure^18^, stroke,^2^ CKD with moderately increased albuminuria, diabetes, metabolic syndrome, cardiovascular diseases,^19^ and left ventricular hypertrophy.^2^

### Clinical approach

When assessing a patient with possible RH, we must consider many important factors to define the diagnostic approach (Table 1).

**Table 1:** Diagnostic approach in resistant hypertension26

Diagnostic Approach
1) Check therapeutic adhesion
2) Rule out pseudo-resistance
3) Adjust anti-hypertensive scheme
4) Perform initial complementary exams (Table 2)
4) Investigate secondary hypertension:
• Obstructive sleep apnea;
• Primary aldosteronism;
• Renovascular hypertension;
• Renal parenchymal disease.
5) Control blood pressure - ABPM

ABPM, Ambulatory Blood Pressure Monitoring.

The first step is to exclude common reasons for pseudo-resistance: inaccurate measurement of BP (special attention should be payed to the adequate size of the cuff for obese patients), poor adherence to both pharmacological and nonpharmacological therapy (i.e. low-sodium diet, physical activity, and weight loss), and an inadequate therapeutic regimen, especially in relation to the use and dosage of the diuretic agents prescribed.^1^^,^^27^^,^^28^ Once the pseudo-resistance is excluded, the following steps are recommended:

#### a) Ambulatory blood pressure monitoring (ABPM)

**Figure 1:**
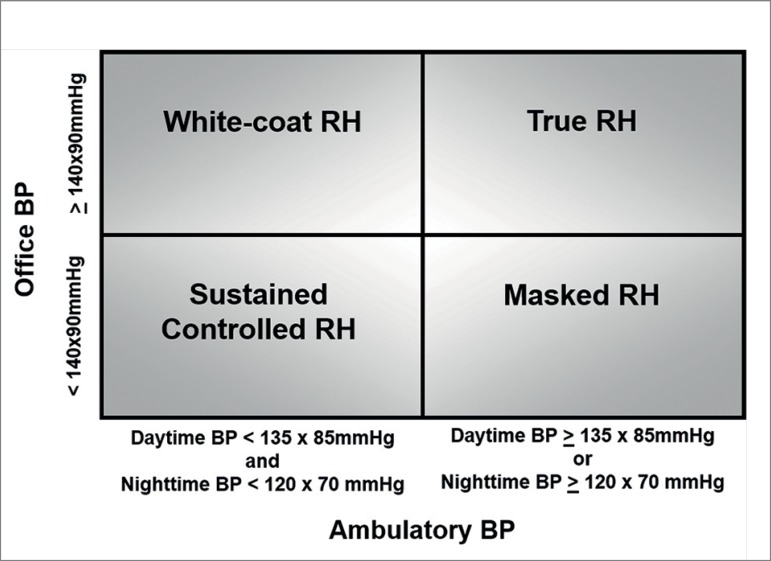
Classification of resistant hypertension into four subgroups according to office and ambulatory blood pressure measurements: controlled, masked, white-coat, and true resistant hypertension (authorized reproduction from Muxfeldt et al^28^)

Even though the definitions of both RH and RfH rely on the office BP measurement higher than 140/90 mmHg, the ABPM is a crucial tool in the diagnosis and follow-up of these patients due to the high prevalence (37% in different series) and magnitude of the white-coat effect observed in these patients.^11^^,^^28^ (Table 2) Moreover, the ABPM allows patients to be classified into 4 distinct groups (Figure 1) that will determine the subsequent diagnostic evaluation and management: true RH (office BP ≥ 140/90 mmHg and either daytime BP ≥ 135/85 mmHg or night time BP ≥ 120/70 mmHg), white-coat RH (office BP ≥ 140/90 mmHg and either daytime BP < 135/85 mmHg and night time BP < 120/70 mmHg), masked RH (office BP < 140/90 mmHg and either daytime BP ≥ 135/85 mmHg or night time BP ≥ 120/70 mmHg), and controlled RH (office BP < 140/ 90 mmHg and either daytime BP < 135/85 mmHg and night time BP < 120/70 mmHg).^11^^,^^28^^,^^29^

**Table 2:** Initial complementary exams

Complementary exams	Indication
ABPM	White coat-effect and nocturnal BP pattern
Fasting plasma glucose/HbA1c	Screening of abnormal glucose tolerance or *diabetes mellitus*
Serum cholesterol, LDL - cholesterol, HDL - cholesterol	Screening of dyslipidemia
Serum uric acid	Monitoring of uric acid by diuretic use. Possible prognostic marker
Serum potassium	Monitoring potassium especially before the onset of spironolactone. Screening of primary aldosteronism
Renal evaluation:	
Serum creatinine	Calculation of estimated GFR (MDRD ou CKD-EPI)Available in: http://ckdepi.org/equations/gfr-calculator/
Urine analysis	Verification of urinary sediment
Urinary protein, creatinine and albuminuria	Calculation of protein/creatinine or albumin/creatinine ratio - asymptomatic target organ or established kidney diseases evaluation
Renal ultrasound	Verification of anatomical changes
12-lead ECG	Screening of left ventricular hypertrophy (voltage criteria and strain pattern)

Notes: ABPM, Ambulatory Blood Pressure Monitoring; HbA1c, Glycated haemoglobin; ECG, electrocardiogram; GFR, glomerular filtration rate.

On the other hand, among RfH patients, the white-coat phenomenon has not yet been adequately studied. In an analysis of the Spanish ABPM Registry^20^, the prevalence of the white-coat effect was lower among refractory when compared to resistant hypertensives (26.7% *versus* 37.1%, *p* < 0.001). In a recent small prospective study with patients with RfH, a prevalence of only 6.5% was found,^30^ suggesting that this phenomenon is much less common among these patients.

In addition, ABPM is essential in the follow-up of these patients at high cardiovascular risk, since it is the only available tool to assess nocturnal blood pressure. In clinical practice, this information allows adjustments to therapeutic regimens based on chronotherapy.^31^^,^^32^ It is recommended that patients with RH use at least one of their anti-hypertensive drugs at bedtime.^31^^,^^32^ It has been demonstrated that chronotherapy was capable of reversing the non-dipper pattern in these patients.^33^

It is known that the non-dipper status is the most common pattern among patients with resistant hypertension, affecting up to 65% of these patients.^11^ Furthermore, it is considered an important prognostic marker, especially for coronary artery disease.^34^ In addition, ambulatory blood pressure during the three periods, but especially at nighttime, are strong predictors of stroke.^35^

The Spanish ABPM registry compared resistant with refractory hypertensives and identified higher ambulatory BP levels in the latter group, with a smaller nocturnal BP reduction. The prevalence of the non-dipper and of the riser patterns was 42.7% and 19.3% among RH patients and of 45.2% and 26.0% among refractory hypertensives, respectively.^20^

#### b) Laboratory exams

At first evaluation, it is necessary to assess the metabolic profile and the renal function (serum creatinine, calculation of the eGFR, and albuminuria dosage) (Table 2).

Patients with RfH have a higher prevalence of diabetes *mellitus* (48.1% *versus* 33.5%, *p* < 0.001) and dyslipidemia (61.9% *versus* 51.7%, *p* < 0.001) than patients with RH.^20^ The association between CKD and RH is well established, as both a cause and a consequence of therapeutic failure. Besides, moderately increased albuminuria and a reduction in the GFR^36^ identify patients with a high cardiovascular risk^37^^,^^38^ and albuminuria reduction may be used as a therapeutic goal in these patients.^39^

A higher prevalence of a eGFR < 60 mL/min/1.73 m^2^ (32.1% *versus* 23.6%, *p* < 0.001) and of moderately increased albuminuria (38.3% *versus* 24.5%, *p* < 0.001) was identified in RfH patients when compared to resistant hypertensives in an analysis of the Spanish ABPM registry.^20^

#### c) Electrocardiogram (ECG)

The 12-lead ECG is a useful tool of low-cost and widely available even in primary health units (Table 2). Left ventricular hypertrophy identified on ECG is an important prognostic marker indicating that a subclinical lesion is under development, even in patients who seem to have a well-controlled office BP. These patients may be experiencing masked RH or isolated nocturnal RH.^8^^,^^9^ The diagnosis of LVH will guide the choice of the therapeutic regimen. Preferentially, an inhibitor of the RAAS should be chosen, aiming for the regression of the LVH.^40^ The electrocardiographic diagnosis of LVH implies an increase in the cardiovascular risk and its prevention or regression aims to improve the prognosis.^40^

On the Spanish ABPM registry, electrocardiographically-diagnosed LVH was more prevalent among patients with RfH than in those with RH (27.6% *versus* 14.9%, *p* < 0.001).^20^

#### d) Causes of secondary hypertension

By definition, in all individuals with suspected RH or RfH, secondary hypertension should be excluded. The most prevalent causes are obstructive sleep apnea, primary aldosteronism, renovascular hypertension, renal parenchymal disease, and pheochromocytoma (Table 3).^41^ Other causes as coarctation of the aorta, Cushing's syndrome, hypo or hyperthyroidism, and acromegaly should be investigated only in situations where there are stigmas of the disease.

**Table 3:** Screening for secondary causes of hypertension

Clinical findings	Suspected diagnosis	Additional investigation
Snore, diurnal somnolence, metabolic syndrome	Obstructive sleep apnea	STOP-BANG questionnaire, Epworth Somnolence Scale. Gold standard: Polysomnography (AIH > 5/hour; moderate apnea: AIH > 15/hour; severe apnea: > 30/hour)
Resistant hypertension with or without hypokalemia. Adrenal nodule	Primary aldosteronism or adrenal hyperplasia	Serum aldosterone > 15 ng/dL Aldosterone/renin ratio > 30 Confirmatory tests: fludrocortisone suppression or saline infusion. Helicoidal CT or MRI
Oedema, anorexia, fatigue, anemia, increased serum urea and creatinine, urinary sediment or anatomic changes	Renal parenchymal disease	Urinalysis, calculation of eGFR, renal ultrasound, urinary albumin/creatinine and protein/creatinine ratio
Abdominal bruit, flash pulmonary oedema, rapid deterioration in renal function after inhibitor of RAAS use.	Renovascular diseases	Renal Duplex Doppler Ultrasonography and/or Magnetic resonance angiography, spiral computed tomography, intra-arterial digital subtraction angiography.
Episodic or persistent high BP with headache, heavy sweating, and palpitations	Pheochromocytoma	Plasma and 24-hour catecholamines and/or metanephrines CT and MRI

AIH, apnea-hypopnea index; CT, computed tomography; eGFR, estimated glomerular filtration rate; MRI, magnetic resonance imaging; RAAS, renin-angiotensin-aldosterone system.

Adapted from Malachias MVB et al. 7ª Diretriz Brasileira de Hipertensão Arterial.^9^

### Therapeutic approach

#### Nonpharmacological strategies

Obesity, as well as physical inactivity, high sodium intake, smoking, and alcoholism are strongly associated with anti-hypertensive treatment failure, all of them considered important risk factors for RH.^1^^,^^11^^,^^34^^,^^42^ In this way, it is imperative to reinforce the importance of lifestyle changes:^8^

- Reduction of dietary sodium intake: (below 2 g/day of sodium, corresponding to 5 g/day of salt);
- DASH diet: use of the Dietary Approaches to Stop Hypertension;^43^
- Weight loss: preferentially a BMI < 25 kg/m^2^;
- Physical activity: practicing aerobic exercises, dynamic resistance training, and isometric resistance training weekly (at least 30 minutes on 5-7 days per week), after cardiology evaluation;
- Smokers: quitting smoking, preferentially with assistance;
- Alcohol: reduce the consumption;
- Avoidance of drugs that increase blood pressure.

#### Pharmacological strategies

The initial cornerstone of resistant hypertension treatment is the association of at least three classes of different drugs: i) an appropriate diuretic, preferentially a long-action thiazide diuretic (ex. chlorthalidone) in patients with normal renal function, or loop diuretics should replace thiazides if eGFR is < 30 mL/min/1.73m^2^ or in other edematous state; ii) a RAAS inhibitor (angiotensin-converting enzyme inhibitors and angiotensin receptor blockers; iii) long-acting dihydropyridines calcium-channel blockers.^1^^,^^8^^,^^9^^,^^27^ Even though hydrochlorothiazide is the most widely prescribed diuretic, chlorthalidone is the diuretic of choice because of its long-acting effect with higher efficacy.^8^^,^^9^ For patients with CKD stage 4 or 5 (eGFR lower than 30 mL/min/1.73m^2^), loop diuretics must be prescribed and administered at least twice a day.^8^^,^^9^

Coronary artery diseases, heart failure, and arrhythmias are special situations when beta-blockers can substitute calcium antagonists at the initial therapeutic scheme with 3 drugs.^8^^,^^9^^,^^27^

RH treatment should be based on diuretic therapy intensification, with special emphasis in the use of spironolactone as a fourth drug, because its association with thiazides provides additive effect in reducing BP.^6^^,^^7^^,^^18^^,^^44^ The ASPIRANT Trial^45^^-^47 showed that the addition of spironolactone (25 mg/day) *versus* placebo lowers systolic BP significantly, especially in older patients. Even in resistant hypertensives with CKD, the spironolactone may be used, except in cases of hyperkalemia.^48^^,^^49^

Recently, the ReHOT study - a Brazilian multicenter study comparing spironolactone *versus* clonidine as a fourth-drug therapy in RH - found that both drugs achieved office and ambulatory BP control in similar rates, but spironolactone promoted greater decreased in systolic and diastolic 24-hour BP and diastolic daytime BP, without nighttime BP difference. Nevertheless, spironolactone was considered preferable as the fourth-drug therapy because of its easier posology, less adverse effects, and consequently better long-term adherence.^50^

If after the four-drug scheme ambulatory BP remains uncontrolled, a fifth-line drug should be added. Possible fifth or sixth drugs are beta-blockers (preferentially the ones with vasodilation effect, as carvedilol, bisoprolol^51^ or nebivolol), central alfa1-agonists (clonidine or doxazosin^51^), and direct vasodilators (hydralazine or minoxidil). The latter two are capable to lowering BP although they do not reduce cardiovascular morbidity and mortality.^8^^,^^9^

Regarding RfH with failure in controlling BP despite the use of optimized therapeutic scheme with 5 or even 6 drugs, new interventions have emerged, as sympatholytic therapies.^18^^,^^24^ Among these new strategies, we highlight the following:

#### Baroreflex activation therapy

The Rheos system is a programmable device that consists of a battery-powered implantable generator that works by electrically activating the carotid baroreflex. The Rheos Pivotal Trial did not identify long-term benefits.^52^

#### Renal sympathetic denervation

The renal denervation procedure uses radiofrequency energy to ablate the nerves within the main renal arteries. This therapy was evaluated by three studies called SYMPLICITY.^53^ Different meta-analyses, including a Cochrane's revision, showed that the procedure was safe, but did not significantly decrease BP.^54^^-^56 The authors advised to await further trials with next-generation catheters, longer follow-up and bigger sample sizes, and especially with standardized procedures.^54^

#### Continuous positive airway pressure (CPAP)

Although the benefits in BP control with CPAP use in resistant hypertensives with moderate-severe sleep apnea are not well established with controversy results in different populations,^57^ the CPAP should be indicated as an adjuvant treatment, in so far as the adherence is greater than 4 hours per night, improving the quality of life and probably reestablishing the dipper pattern.^58^

#### Central Iliac arteriovenous anastomosis

The ROX Medical arteriovenous coupler is a stent-like device that exhibits shape memory to self-expand, forming an AV anastomosis in central iliac. The ROX control HTN demonstrated significant BP decrease, possibly reducing cardiovascular morbidity in those patients.^59^ Notwithstanding, this is an isolated study and more clinical evidence is necessary.

Table 4 summarizes the main differences between resistant and refractory hypertension observed in various populations.

**Table 4:** Characteristics of resistant and refractory hypertension

Characteristics	Resistant hypertension	Refractory hypertension
Prevalence	10-20%	5%
Mechanism	Volume-dependent	Increased sympathetic activity
Gender	Women	Women
Age	Older	Younger
Obesity	↑	↑↑
Diabetes	↑	↑↑
Dyslipidemia	↑	↑↑
Left ventricular hypertrophy	↑↑	↑↑↑
Moderately increased albuminuria	↑	↑↑
eGFR < 60 ml/min/1,73m^2^	↑	↑↑
Coronary heart disease	↑	↑
Previous cardiovascular disease	↑↑	↑↑↑
Obstructive sleep apnea	↑	Undetermined
Aldosterone	↑	↔
Sodium	↑	↔
Cardiovascular risk	↑↑	Apparently increased

eGFR, estimated glomerular filtration rate.

## Conclusion

Despite the common final pathway of hypertension encompassing hydrosaline retention and increased sympathetic tonus, the existence of many phenotypes with distinct clinical paths and prognosis, a broad spectrum ranging from easily controlled hypertension to RH, and more recently, RfH has been suggested.

Even though these two entities are frequently considered a continuum of the same process, it is interesting to observe that they seem to have different pathophysiological mechanisms, suggesting two distinct conditions.

RH patients compared with general hypertensives, are older and more obese. The principal associated comorbidities are established CKD, diabetes, sleep apnea, stroke, and cardiovascular diseases, all of them involving the hyperaldosteronism status. ABPM is mandatory in the diagnosis and follow-up of those patients because of a high magnitude of the white-coat effect.

Moreover, refractory hypertensives compared with controlled RH are younger, predominantly women, and Afro-american. They also have a high prevalence of heart failure, stroke, and CKD with moderately increased albuminuria and LVH. The white-coat effect seems to be less evident in those patients.

In RH, the therapeutic scheme should be based on the intensification of diuretic therapy, emphasizing the spironolactone as the fourth drug associated with a long-acting thiazide, as chlorthalidone. On the other hand, as RfH usually fails all used classes of anti-hypertensives including association of different diuretics, RfH treatment is not well established and new therapies have been proposed such as sympatholytic intervention.

The unfavorable cardiovascular and renal prognosis of RH patients is well established, but future longitudinal studies are necessary to define the morbidity and mortality of RfH.

Resistant and refractory hypertension are challenges in clinical practice and should be addressed as different entities, ideally by specialized professionals capable of identifying comorbidities and to provide diversified and individualized treatment.
